# Frailty is associated with low physical activity and poor sleep quality in patients undergoing myeloablative allogeneic hematopoietic cell transplantation: a Fitbit® pilot study

**DOI:** 10.3389/fmedt.2025.1605164

**Published:** 2025-12-08

**Authors:** Caryn R. Libbert, Fiona He, Najla El Jurdi, Helen Fagrelius, Mark Juckett, Joseph Maakaron, William Juckett, Nicholas Evanoff, Donald R. Dengel, Shernan G. Holtan

**Affiliations:** 1 University of Minnesota Medical School, Minneapolis, MN, United States; 2 Allina Health Cancer Institute, Minneapolis, MN, United States; 3 Division of Hematology, Oncology, and Transplantation, Department of Medicine, University of Minnesota, Minneapolis, MN, United States; 4 National Institutes of Health, Bethesda, MD, United States; 5 Clinical Trials Office, Masonic Cancer Center, University of Minnesota, Minneapolis, MN, United States; 6 School of Kinesiology, University of Minnesota, Minneapolis, MN, United States; 7 Roswell Park Comprehensive Cancer Center, Buffalo, NY, United States

**Keywords:** allogeneic hematopoietic cell transplant, frailty, STEPS, sleep, fitbit

## Abstract

**Introduction:**

Frailty, a multidimensional syndrome of reduced physiologic reserve, is associated with poorer outcomes following allogeneic hematopoietic cell transplantation (alloHCT), even among younger adults. This pilot study explores whether wearable sensor data reflecting physical activity and sleep are associated with pre-transplant frailty status in patients undergoing myeloablative alloHCT.

**Methods:**

Adults undergoing first myeloablative alloHCT at the University of Minnesota from June 2022 to January 2023 were enrolled and given Fitbit® Sense devices. Frailty was assessed pre-transplant using Fried Phenotype criteria. Activity and sleep data were collected from hospital admission to day +30 post-transplant. Descriptive and inferential statistics assessed differences across frailty phenotypes.

**Results:**

Nine patients were included: 2 not frail, 5 pre-frail, and 2 frail. Not frail patients demonstrated significantly higher daily steps and active minutes, and lower sedentary time compared to pre-frail and frail groups (all *p* < 0.01). Frail individuals had significantly reduced deep and REM sleep. The nadir for sleep and peak in sedentary behavior occurred around day +15 post-transplant.

**Conclusion:**

Pre-transplant frailty was associated with decreased physical activity and less restorative sleep during the peri-transplant period. These findings support further study of wearable data to guide personalized supportive care strategies in alloHCT recipients.

## 1 Introduction

Allogeneic hematopoietic cell transplantation (alloHCT) is a therapeutic approach for a range of hematologic disorders. It is achieved by replacing diseased bone marrow with hematopoietic stem cells from a healthy donor. Despite its clinical benefits, successful alloHCT requires considerable physiological resilience. Frailty is characterized by diminished physiological reserves, decreased capacity for stress adaptation, and impaired tissue repair. It can manifest in alloHCT candidates across age groups as a result of both underlying disease and treatment-related toxicities. Its presence is associated with elevated risks of mortality and post-transplant complications (1–3). Frailty also exerts significant physical, psychological, and social burdens that compromise overall recovery. In this study, frailty status is systematically assessed at baseline using validated clinical criteria. Specifically, the Fried Frailty Phenotype categorizes participants as not frail, pre-frail, or frail based on standardized measures of grip strength, walking speed, physical activity, exhaustion, and unintended weight loss. To objectively monitor well-being during the peri-transplant period, wearable devices such as Fitbit® devices can be utilized. These devices continuously track daily activity and sleep patterns, which serve as important indicators of patient health (4). Existing evidence already demonstrates a relationship between frailty and poorer alloHCT outcomes. However, the specific activity and sleep trajectories associated with varying degrees of frailty, particularly in adults receiving myeloablative alloHCT who were otherwise functionally fit before transplantation, remain insufficiently studied. This pilot trial seeks to preliminarily characterize these patterns, thereby providing context informing future individualized supportive care studies. Such interventions may enhance patient quality of life and post-transplant survival.

## 2 Patients and methods

### 2.1 Patient selection

We included adults (≥18 years) scheduled for their first myeloablative alloHCT at the University of Minnesota who had a smartphone or tablet compatible with the Fitbit® app from June 2022 to January 2023. Participants received a Fitbit® Sense device before their alloHCT hospitalization and were advised to stay active by walking as desired, without specific restrictions, within the hospital unit. However, patients typically remained in their rooms during chemotherapy or blood product administration. Access to physical therapists and exercise facilities were provided. The study adhered to IRB approval and HIPAA guidelines, with all participants giving written informed consent prior to initiating study-related activities.

### 2.2 Data collection and definitions

Participants synced their Fitbit® with the private health information-compliant Fitabase® at least weekly. Data from hospital admission until day +30 post-transplant, considering days with ≥10 h of wear time as evaluable days, were included in this analysis. Activity was measured via daily steps, active and sedentary time—active time being periods with ≥3 metabolic equivalents (METS) energy expenditure. Sleep analysis included the categories of overall duration, deep sleep, and REM sleep. Frailty status, assessed pre-transplant using Fried Phenotype criteria (5), categorized individuals as frail (≥3 criteria), pre-frail (1–2 criteria), or not frail (no criteria), with data extracted from health records where frailty was formally assessed by a trained clinician prior to transplant.

### 2.3 Statistical methods

Descriptive statistics of daily steps, active minutes, sedentary minutes, minutes asleep, minutes in deep sleep, and minutes in REM sleep were analyzed by frailty status. Differences between not frail, pre-frail, and frail groups were analyzed using Kruskal–Wallis tests and Dunn's multiple comparisons for *post hoc* assessments. Smoothing spline regression curves were generated using data from all patients to demonstrate longitudinal activity and sleep trends (*λ* = 100) across the entire cohort. Statistical analyses were completed using JMP Pro 17 (SAS Corporation, Cary, NC) and GraphPad Prism version 9.4.1 (GraphPad Software, Boston, MA).

## 3 Results

### 3.1 Patient characteristics

Nine patients with adequate data were included in the analysis. The cohort is balanced by sex (5 men, 4 women) with a median age of 48 years (range 22–59 years). Underlying diagnoses were dominated by acute leukemias (6 of 9: 3 acute myeloid leukemia, 3 acute lymphoblastic leukemia), and donor sources are split between matched related (4) and matched unrelated (5). Most patients were pre-frail (5 of 9), with 2 frail and 2 not frail. In-hospital complications were frequent, nearly universal mucositis and common nutrition support with total parenteral nutrition, yet day−100 outcomes show 7 of 9 relapse-free, with one relapse (alive) and one relapse-related death. Additional patient characteristics are included in Supplementary Table S1. The mean percentage of evaluable days between hospital admission to day +30 was 81% ± 22% with a mean wear time of 21.8 ± 3.3 h.

### 3.2 Patterns of activity

**Figure 1:**
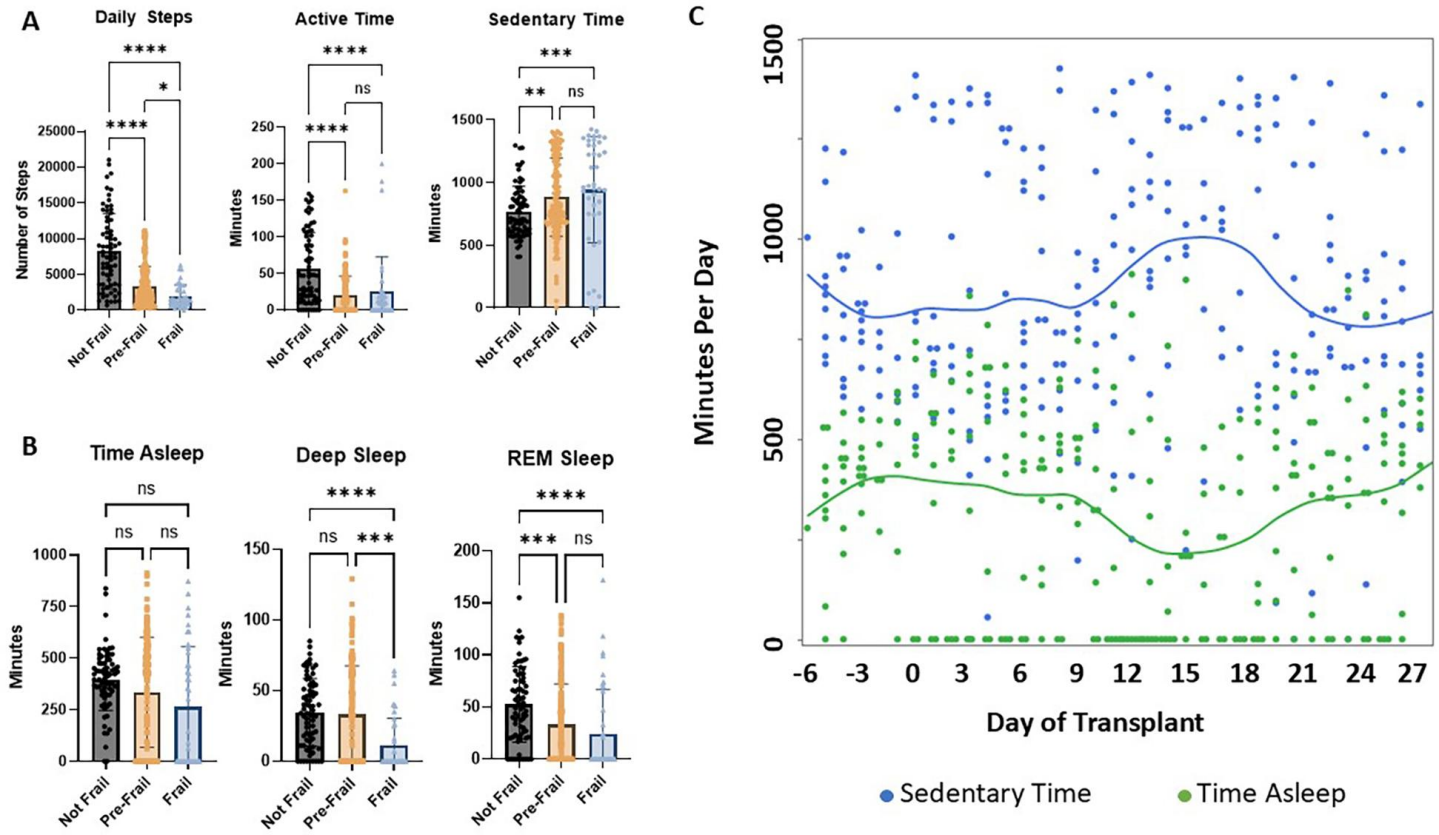
Patterns of **(A)** activity and **(B)** sleep based on phenotype of frailty. The top of the bar represents the mean with error bars representing standard deviation. Longitudinal sedentary time and minutes asleep by transplant date are shown in **(C)**, with the line representing the smoothing spline regression curves. Each dot corresponds to the data point of an individual participant on a single evaluable day. Significance levels are as follows: *p < 0.05, **p < 0.01, ***p < 0.001, ****p < 0.0001.

Not frail patients had a higher mean of daily steps compared to pre-frail (2.4-fold higher, 8265 ± 5,274 vs. 3,381 ± 2,757 steps, *p* < 0.0001) and frail (4.2-fold higher, 1,977 ± 1,610 steps, *p* < 0.0001). Pre-frail patients had a higher mean of daily steps than frail (1.7-fold higher, *p* = 0.0103, Figure 1A). Not frail patients also had a higher mean of active minutes compared to pre-frail (56 ± 49 vs. 20 ± 26 min, *p* < 0.0001) and frail (25 ± 47 min, *p* < 0.0001). There was no significant difference in the mean of active minutes between pre-frail and frail patients (*p* > 0.9999). Not frail patients had a lower mean of sedentary minutes compared to both pre-frail (763 ± 206 vs. 884 ± 313 min, *p* = 0.0064) and frail (943 ± 422 min, *p* = 0.0004). There was no statistically significant difference in the mean sedentary time between pre-frail and frail patients (*p* = 0.2472). Maximum sedentary time across the cohort occurred around day +15 post-transplant (Figure 1C).

### 3.3 Patterns of sleep

Across all cohorts, there was a median percentage of 67% (range 0% to 100%) of evaluable days including complete sleep data with sleep stages from admission to day +30. There was no statistically significant difference in mean minutes asleep between not frail and pre-frail (*p* > 0.9999), not frail and frail (*p* = 0.0904), or prefrail and frail (*p* = 0.2601, Figure 1B). However, frail patients spent less time in deep sleep compared to pre-frail (11 ± 19 vs. 33 ± 34 min, *p* = 0.0003) and not frail (35 ± 24 min, *p* < 0.0001). There was no statistically significant difference in mean minutes spent in REM sleep between pre-frail and frail patients (*p* = 0.1793). Not frail patients spent more time in REM sleep compared to pre-frail (52 ± 36 vs. 33 ± 39 minutes, *p* = 0.0003) and frail (24 ± 43 min, *p* < 0.0001). There was a median percentage of 28% (range 0% to 58%) of days with zero minutes of recorded time asleep, a median percentage of 36% (range 3% to 87%) of days with zero minutes of time in deep sleep, and a median percentage of 36% (range 0% to 87%) of days with zero minutes of REM sleep as measured by the Fitbit®. The sleep time nadir across the cohort was also around day +15 post-transplant (Figure 1C).

## 4 Discussion

Our study preliminarily describes the variances in activity and sleep patterns among three distinct frailty phenotypes prior to transplantation. We discovered that patients without frailty exhibit higher daily step counts and more active time than their pre-frail and frail counterparts. Furthermore, individuals classified as frail not only engaged in less physical activity but more surprisingly also experienced diminished durations of restorative deep and REM sleep. A median of 28% of the days without recorded sleep does not mean that sleep did not occur. Rather importantly, the nights of “zero sleep” reflect device scoring rules (≥60 min of immobility to log an episode; ≥3 h to stage sleep) (6, 7). This information is not truly missing data; rather it may be highlighting the poor quality of sleep many patients may encounter in the hospital. Fitbit® sleep staging has not been validated for acutely ill inpatients with frequent overnight interruptions, and our findings should be interpreted within that constraint.

Notably, the peak in sedentary time and nadir in device-scored sleep around day +15 likely reflects convergent clinical and operational factors typical of the pre- to peri-engraftment period. At this time, cytopenia-related care (e.g., transfusions, fever evaluations, antimicrobial changes) increases nighttime interruptions; mucositis and gastrointestinal toxicity drive analgesic and antiemetic use; and early engraftment inflammatory phenomena may disrupt sleep architecture. In addition, substantial pre-engraftment fatigue promotes daytime bedrest. These conditions fragment sleep while simultaneously increasing daytime sedentary behavior. The occurrence of days without recorded sleep data suggests that sleep disruptions are prevalent across all patient groups. The frequent nighttime awakenings, as reported by El Jurdi et al., averaging 4.5 interruptions per night, are a probable factor in the poor sleep quality captured in our findings (4).

A clear limitation in our study is its small sample size. A small sample size does not, by itself, invalidate a study when the design is explicitly exploratory and the data are unusually rich. Within-subject designs, time-course sampling, and transparent limitations can yield valuable insights even when the sample size is small. The Fitbit® data we collected contains thousands of data points, similar to a gene expression analysis. Even though we were only able to enroll nine patients, the longitudinal pilot data yield unique preliminary insights. Over 80% of our days were evaluable, with over 21 h of wear time on these days, highlighting the rich time coverage of the data collected.

Previous research has linked frailty to decreased overall survival, increased non-relapse mortality, and a decline in the quality of life (2, 3, 8, 9). The Fried Phenotype Criteria (weight loss, exhaustion, weakness, slowness, and low physical activity) offer a practical framework for identifying pre-frail and frail individuals using simple, reproducible measures (5). However, its focus on physical domains may underestimate cognitive, psychological, or social concerns, and transient treatment-related effects (such as anemia or deconditioning during allo-HCT), limiting precision for dynamic inpatient settings. Our observed reduced activity and lack of restorative sleep observed in frail patients are likely contributing to these negative health outcomes. Interventions tailored to the specific aspects of frailty in the elderly have been shown to enhance physical condition, as noted by Cameron et al. (10). Examples of tailored components include dietitian-guided supplementation and meal support for weight loss, psychology referral and social-engagement for exhaustion, and progressive strength programs coordinated by physical therapists for slowness/weakness, delivered within a case-managed framework. While effective for frailty, such multifactorial models are resource-intensive and adherence-dependent. Nonetheless, this insight opens the possibility of applying similar targeted interventions to alloHCT patients, employing pre-transplant frailty assessments to inform and customize therapeutic strategies, potentially mitigating the impact of frailty on post-transplant recovery and long-term well-being.

## 5 Conclusion

Our pilot study preliminarily demonstrated a reduced level of physical activity and poor sleep in individuals with frailty undergoing myeloablative alloHCT. Recognizing these differences may allow for development of targeted interventions to reduce disease burdens of care and improve support systems.

## Data Availability

The datasets presented in this article are not readily available because requests for data may be made to the corresponding author. Requests to access the datasets should be directed to sghmd@pm.me.
