# Supplementary material for: Frailty is associated with low physical activity and poor sleep quality in patients undergoing myeloablative allogeneic hematopoietic cell transplantation: a Fitbit® pilot study
Source: Front Med Technol. 2025 Dec 8;7:1605164. doi: 10.3389/fmedt.2025.1605164 (PMC12719429; doi:10.3389/fmedt.2025.1605164)
Supplement: Supplementary file 1 [file Datasheet1.pdf]

Participant	Diagnosis	Age (years)	Sex	Donor type	Frailty status with criteria met	Fat/Lean Mass Ratio	Bone Density	Length of hospitalization (days)	Complications during hospitalization	Day 100 relapse and mortality outcomes
1	Mantle cell lymphoma	46	M	Matched related donor	Not frail	0.249	1.335	24	▪Mucositis<br>▪Malnutrition requiring total parenteral nutrition	Relapse free
2	Acute myeloid leukemia	22	M	Matched related donor	Frail<br>▪Weight loss<br>▪Exhaustion<br>▪Weakness	0.380	1.123	27	▪Mucositis<br>▪Malnutrition requiring total parenteral nutrition	Relapse-related mortality
3	Acute lymphoblastic leukemia	59	F	Matched unrelated donor	Pre-frail<br>▪Weight loss<br>▪Weakness	0.697	1.098	36	▪Mucositis<br>▪Malnutrition requiring total parenteral nutrition	Relapse free
4	Chronic myeloid leukemia	48	M	Matched unrelated donor	Not frail	0.268	1.169	25	▪Mucositis<br>▪Neutropenic fever	Relapse free
5	Acute myeloid leukemia	57	F	Matched unrelated donor	Pre-frail<br>▪Exhaustion<br>▪Weakness	0.468	0.956	25	▪Mucositis<br>▪Malnutrition requiring total parenteral nutrition<br>▪Clostridium difficile	Relapse free

6	Acute lymphoblastic leukemia	28	M	Matched related donor	Pre-frail<br>▪Weakness	0.474	1.096	33	▪Mucositis<br>▪Neutropenic fever<br>▪Non-neutropenic fever of unknown source<br>▪Anterior mediastinal hematoma<br>▪Malnutrition requiring total parenteral nutrition	Relapse free
7	Myelodysplastic syndrome	57	F	Matched unrelated donor	Pre-frail<br>▪Weakness<br>▪Slow walking speed	0.564	1.042	27	▪Mucositis<br>▪Neutropenic fever	Relapse, alive
8	Acute lymphoblastic leukemia	38	M	Matched related donor	Frail<br>▪Weight loss<br>▪Weakness<br>▪Low physical activity	0.502	1.113	24	▪Mucositis<br>▪Neutropenic fever<br>▪Bacteremia	Relapse free
9	Acute myeloid leukemia	53	F	Matched unrelated donor	Pre-frail<br>▪Exhaustion<br>▪Weakness	0.539	1.026	27	▪Mucositis<br>▪Neutropenic fever<br>▪Malnutrition requiring total parenteral nutrition	Relapse free
